# Burden of metabolic syndrome among primary care patients in Crete, Greece: A descriptive study

**DOI:** 10.1080/13814788.2020.1851676

**Published:** 2020-12-15

**Authors:** Marilena Anastasaki, Sophia Papadakis, Manolis Linardakis, Dimitrios Anyfantakis, Emmanouil K. Symvoulakis, Christos Lionis, on behalf of the Cretan Primary Care Research Group

**Affiliations:** Faculty of Medicine, Clinic of Social and Family Medicine, University of Crete, Crete, Heraklion, Greece

**Keywords:** Metabolic syndrome, cardiovascular disease, risk factors, primary care, Greece

## Abstract

**Background:**

Metabolic Syndrome (MetS) is a clustering of abdominal obesity, hypertriglyceridaemia, low HDL cholesterol, hyperglycaemia and hypertension. Early identification of MetS is important for preventing cardiovascular disease (CVD). MetS has not been systematically explored in Greek primary care.

**Objectives:**

To examine MetS frequency among primary care patients 40 years of age or older in Crete.

**Methods:**

A descriptive study was conducted (July–December 2015). General practitioners, randomly selected from regional physician listings, recruited consecutive patients, 40 years of age or older, visiting their practice. Chart audits were completed for eligible patients using medical records, including demographics and cardiovascular risk factors (hypertension, dyslipidaemia, diabetes mellitus). MetS was defined using the revised NCEP ATP III criteria. Frequencies with 95% confidence intervals were calculated. Gender differences were explored using Chi-square and Mann–Whitney tests.

**Results:**

Our sample consisted of 815 patients (55.7% female; mean age 65.2 years; range 40–98 years) from 44 GP practices. Overall, 73.6% (95% CI 70.4, 76.6) were identified with MetS, with a higher proportion of males (75.6%; 95% CI 71.0, 79.8) than females (72.0%; 95% CI 67.8, 76.0). Among the total sample, relatively high rates of hypertension (males: 64.5%; 95% CI 59.9, 70.0 and females: 61.1%; 95% CI 56.8, 65.8), dyslipidaemia (males: 69.3%; 95% CI 64.3, 74.1 and females: 63.5%; 95% CI 59.3, 68.0), diabetes mellitus (males: 46.9%; 95% CI 42.2, 52.4 and females: 36.5%; 95% CI 32.5, 41.6) and coronary heart disease (males: 21.2%; 95% CI 17.0, 25.2 and females: 6.2%; 95% CI 4.2, 8.6) were documented.

**Conclusion:**

MetS and CVD risk factors were encountered at high frequencies in the studied population of primary care patients in Crete.

KEY MESSAGES

- High frequencies of MetS (73.6%) were documented in our primary care population of adults aged 40 years or older.
- Frequencies of hypertension, dyslipidaemia, diabetes and coronary heart disease (CHD) were high, with significant gender differences for diabetes and CHD.
- Additional research is necessary to validate these findings.

## Introduction

Metabolic Syndrome (MetS) is defined as a clustering of interconnected cardiovascular disease (CVD) risk factors, namely abdominal obesity, hypertriglyceridaemia, low HDL cholesterol, hyperglycaemia and hypertension [1]. Evidence suggests that patients with MetS have increased risk of developing CVD [2], particularly Coronary Heart Disease (CHD) [3]. MetS is considered a major healthcare issue of western societies, reaching epidemic proportions in many countries [4].

Clinical guidelines recommend primary care providers assess MetS and its related risk factors in patients over 40 years old [5,6]. However, MetS data in Greece are significant. Results of a population-based study indicated a MetS prevalence of 19.8% [7]. Additionally, a large-scale study in a representative sample of the Greek adult population documented a high, age-standardised MetS prevalence of 24.5% [8]. Despite the availability of population-based prevalence data, there is lack of evidence regarding the prevalence of MetS in primary care practice settings in Europe, including Greece.

Internationally, there is an active discussion among several medical disciplines about enhancing the role of General Practitioners (GPs) in MetS care and CVD prevention [9]. Health reforms are currently unfolding in Greece to improve quality and effectiveness of primary care and data on MetS and CVD risk factors’ frequencies is necessary for optimising primary care delivery according to patients’ needs. To this end, this study aimed to identify the burden of MetS and provide evidence regarding the frequency of CVD risk factors among patients 40 years of age or older, visiting primary care practices in Crete, Greece.

## Methods

### Design and setting

A descriptive study of patients from a random sample of primary care practices in the island of Crete, Greece was conducted to assess the burden of MetS and CVD risk factors. ‘Burden’ is defined as the number of patients with METS/CVD risk factors in the population of patients visiting their GP (in a given period), i.e. the frequency with which the GP is confronted with METS/CVD risk factors during consultation hours (in a given period) and is distinguished from the traditional concept of ‘prevalence’ (i.e. the number of known patients with METS/CVD risk factors present in the GP’s entire practice population at a certain moment).

Two-stage sampling was performed. First, stratified random sampling was used to select 30% of the overall number of GPs currently practising in Crete (*n* = 250). Stratification was based on geographic district (four counties) and area of practice (urban/rural, ‘rural’ defined as any area outside each county’s capital). Using available registries, a total of 77 was selected. In the second stage, each participating GP targeted to enrol 25 eligible patients.

### Participants

Patient recruitment took place during July–December 2015. Participating GPs screened and recruited patients consecutively visiting their practices during the recruitment. Patient inclusion criteria were: 1) aged 40 years or older, 2) permanent resident of each district and 3) availability of data within the past three months in electronic or paper-based records for at least three of the following laboratory tests or measurements: HbAc1, fasting glucose, HDL cholesterol, total serum cholesterol, fasting triglycerides, systolic and diastolic blood pressure or CRP. Criterion 1 was applied for identifying the burden in an age category where secondary prevention is effective. Criterion 2 was applied to ensure the study reflects the needs of the local population. Criterion 3 was applied to enable MetS classification based on available data. Exclusion criteria were the counterparts of inclusion criteria.

The study was approved by the Bioethics Committee of the 7th Health Region of Crete (Pr. No: 2774/14-5-2015). All participants had to be able to sign their informed consent form prior to inclusion, which included a statement regarding the anonymous use of their data for the present study only. No consent forms signed by other individuals on behalf of any participant were accepted.

### Measurements

Eligible patients were invited to complete a questionnaire, assisted by their GP. Questions assessed factors that may not have been available in Greek primary care records, including socio-demographic characteristics (age, gender, education, income, area of residence, marital status), smoking and physical activity (i.e. at least 20 min of vigorous activities three times per week). GPs retrieved information about laboratory tests (HDL/LDL cholesterol, triglycerides, fasting glucose), co-morbidities and prescriptions for MetS-relevant conditions from patients’ records (no new tests were performed). Body measurements (weight, height, waist circumference) and blood pressure were recorded upon physical examination conducted by GPs. When the researchers identified missing data, contact with the GP was attempted to retrieve information.

Patients were classified using the revised National Cholesterol Education Program’s Adult Treatment Panel III (NCEP ATP III) MetS definition for [10]. This definition was selected for reasons of comparison with other Greek studies and since it provides the simplest MetS classification method. MetS was identified if three or more of the following risk factors were present:

- Abdominal obesity: elevated waist circumference: ≥102 cm for males and ≥88 cm for females.
- Dyslipidaemia: elevated triglycerides: ≥150 mg/dL or on drug treatment for elevated triglycerides.
- Dyslipidaemia: Reduced HDL cholesterol: <40 mg/dL for males and <50 mg/dL for females or on drug treatment for reduced HDL cholesterol.
- Hypertension: systolic blood pressure: ≥130 mmHg or diastolic blood pressure ≥85 mmHg or on antihypertensive drug treatment in a patient with a history of hypertension.
- Hyperglycaemia: elevated fasting glucose: ≥100 mg/dL or on drug treatment for elevated glucose.

### Sample size

Sample size calculations assumed no effect of provider level clustering, a 50% MetS frequency among patients meeting the inclusion criteria [8] and the following parameters: *p* = 0.245, 1-*p* = 0.755, type-I error probability *a* = 0.10 (or 90% power), precision *d* = 0.02 and population of 600,000 people (population of Crete) [11]. The required sample size was estimated at 1250 patients [12].

### Statistical analysis

Data were analysed using SPSS software (IBM SPSS Statistics, Version 25.0. Armonk, NY, USA: IBM Corp). Descriptive statistics were calculated for MetS frequencies, risk factors and cardiometabolic conditions, with 95% confidence intervals (95% CIs) estimated by bootstrap techniques. MetS frequencies were calculated by dividing the number of participants identified with three or more risk factors or MetS by the total sample size achieved. Gender and age differences were explored through Chi-square and non-parametric Mann–Whitney tests. Critical *p*-value was 0.05. No data imputation was used for missing data. If data required for Mets calculation was missing, the case was excluded from the denominator and overall analysis.

## Results

### Participants

**Figure 1.**
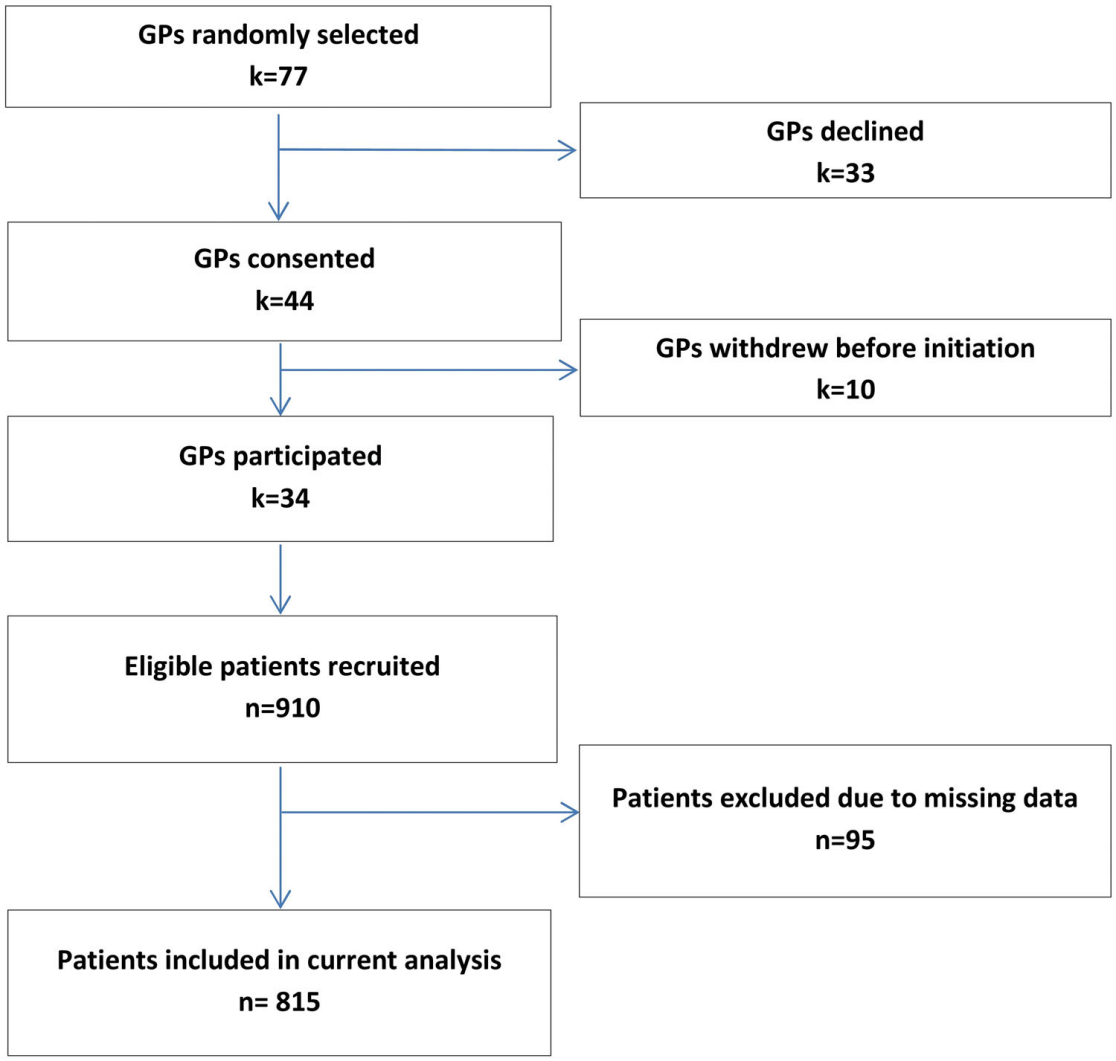
Recruitment flowchart. k: general practitioners; *n*: patients.

Figure 1 shows the study flow diagram. Of the 77 randomly selected GPs, 34 agreed to participate (23 from urban and 11 from rural practices). There were no significant differences in GPs’ response among strata (Chi-square tests’ *p*-values > 0.10). GPs recruited 910 patients, of which 95 were excluded due to missing data. The average number of recruited patients per GP (after exclusion of cases with missing data) was 24 (range: 5–35), denoting that some practices recruited less and others more than the target of 25 visitors. Data from 815 patients were finally included in this analysis.

### Socio-demographic characteristics

Table 1 presents patients’ socio-demographic characteristics and health-related behaviours. The sample included a greater proportion of females (55.7%) than males (44.3%). Mean age was 65.2 years (SD ± 12.0, range: 40–98). The majority was living in urban areas (63.9%), and had less than high school education (56.7%).

**Table 1.** Socio-demographic characteristics and health habits of 815primary care patients, 40 years of age or older, with availability of recent laboratory examinations. Crete (Greece), 2015.

Variable	*n*	%
Gender		
Male	361	44.3
Female	454	55.7
Age, years		
40–59	268	33.0
60–79	457	56.4
80–98	86	10.6
mean ± stand. dev. (min.–max.)	65.2 ± 12.0 (40–98)	
Marital Status		
Single	54	6.7
Married	602	74.4
Divorced	28	3.5
Widowed	125	15.5
Area of living		
Urban	521	63.9
Rural	294	36.1
Occupation		
Employed	409	50.2
Unemployed / retired	405	49.8
Education level		
None	79	9.8
Primary school	378	46.9
High school	237	29.4
University, college	102	12.7
Post graduate	5	0.6
PhD	5	0.6
mean years ± stand. dev. (min.–max.)	6.1 ± 4.1 (0–20)	
Monthly family income, Euros		
<500	229	29.3
500–1000	329	42.2
1000–1500	168	21.5
1500–2000	22	2.8
>2000	33	4.2
Smoking		
Current smoker	156	19.2
No/former smoker	658	80.8
Physical activity (at least 20′ of vigorous activities for 3 times/week)		
Yes	203	25.0
No	608	75.0

### MetS-related conditions

**Figure 2.**
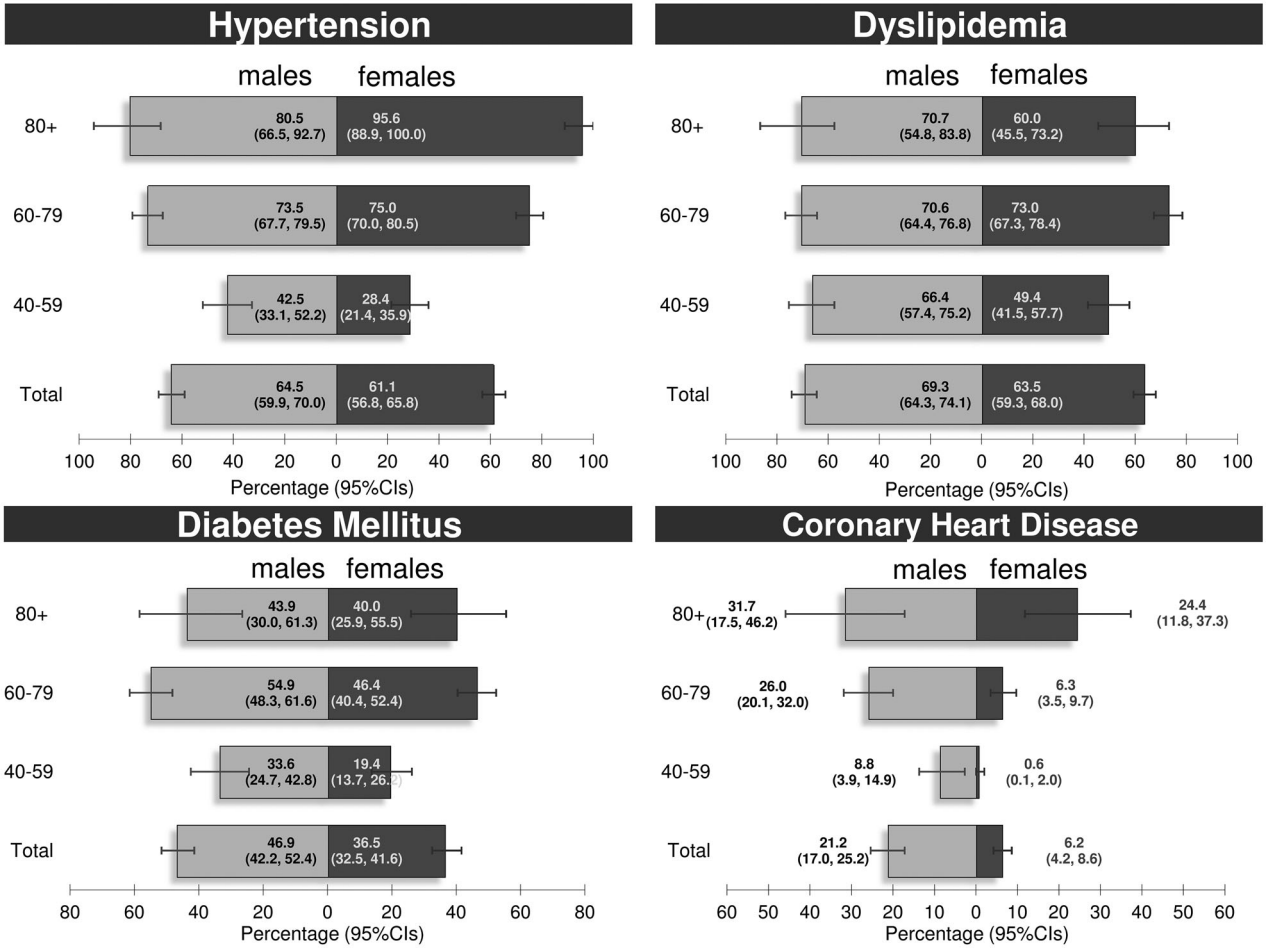
Frequencies of cardio metabolic conditions in primary care patients, 40 years of age or older, with availability of recent laboratory examinations, by gender and age. Crete (Greece), 2015. 810 out of 815 patients had complete data for hypertension, dyslipidaemia, diabetes mellitus, and coronary heart disease. Grey lines show the 95% confidence intervals (95% CIs).

Proportions of patients with hypertension were 64.5% for males and 61.1% for females, while dyslipidaemia accounted for 69.3% of males and 63.5% of females (Figure 2). Frequencies of diabetes mellitus were significantly higher in males than females (46.9 vs. 36.5%; *p* = 0.005). A statistically significant difference in CHD frequencies was observed between males and females (21.2 vs. 6.2%; *p* < 0.001). The frequency of multi-morbidity (3+ diseases) reached 61.7% and was similar in males and females. The Supplementary Table presents medication data for MetS-related conditions in 804 out of 815 patients without missing prescription data. Overall, 64.8, 39.7, and 62.4% of patients were on medication for hypertension, diabetes, and dyslipidaemia, respectively.

### MetS risk factors

Table 2 summarises levels of NCEP ATP III risk factors. Mean values of all MetS factors were close to NCEP ATP III thresholds. Additionally, large proportions of participants had abnormal levels of all MetS factors Compared to females, a higher proportions of males had elevated blood pressure (*p* = 0.031), fasting glucose (*p* < 0.001) and triglycerides (*p* = 0.009), while larger proportion of females had increased waist circumference (*p* < 0.001).

**Table 2.** Frequencies of metabolic syndrome (MetS) risk factors in primary care patients, 40 years of age or older, with availability of recent laboratory examinations. Crete (Greece), 2015.

Risk factors & MetS criteria^a^	Mean (stand. dev.)			*p*-Value ^b^
	Total (*n* = 815)	Males (*n* = 361)	Females (*n* = 454)	
Systolic blood pressure (mm Hg)	129.8 (14.3)	131.2 (12.8)	128.6 (15.2)	0.002
Diastolic blood pressure (mm Hg)	78.5 (9.0)	79.0 (9.1)	78.0 (8.9)	0.183
>130/85 mmHg or medication	72.4%	76.2%	69.4%	0.031
Fasting glucose (mg/dL)	111.7 (33.7)	116.3 (36.7)	108.1 (30.7)	<0.001
≥100 mg/dl or medication	61.8%	71.2%	54.4%	<0.001
Triglycerides (mg/dL)	138.6 (78.5)	144.1 (89.8)	134.2 (68.0)	0.130
≥150 mg/dl or medication	70.3%	75.1%	66.5%	0.009
HDL cholesterol (mg/dL)	51.6 (15.7)	48.3 (14.3)	54.2 (16.3)	<0.001
<40 mg/dl (M), <50 mg/dl (F) or medication	71.4%	69.0%	73.3%	0.185
Waist circumference (cm)	103.2 (15.5)	107.0 (14.2)	100.1 (15.9)	<0.001
>102cm (M), >88cm (F)	66.0%	59.3%	71.4%	<0.001

^a^According to the National Cholesterol Education Program’s Adult Treatment Panel III (NCEP ATP III–revision 2005) guidelines for metabolic syndrome (MetS).

^b^*p*-Values calculated using Mann–Whitney and Chi-square tests.

### MetS burden

**Figure 3.**
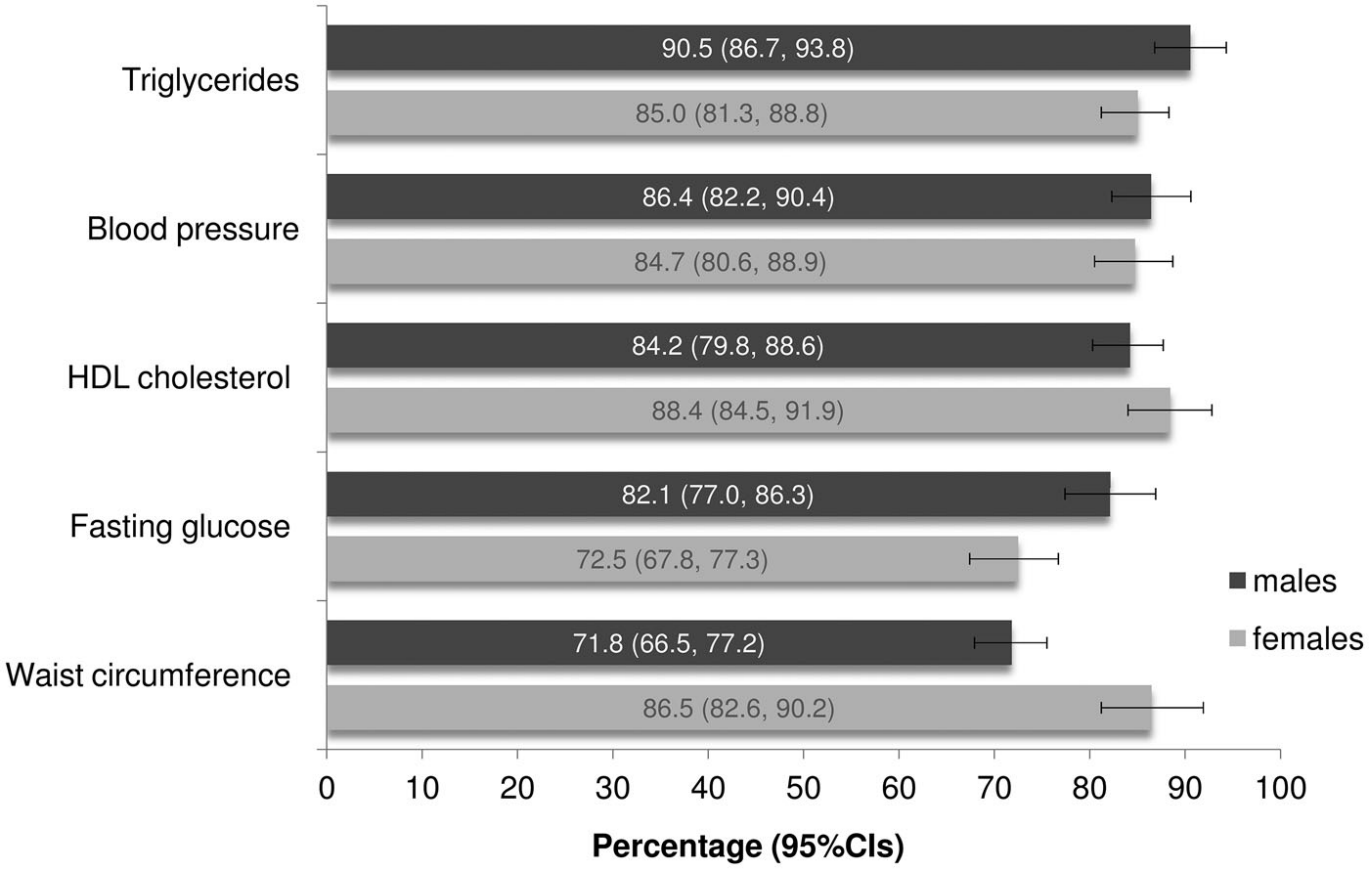
Gender differences in risk factor frequencies among patients 40 years of age or older, with availability of recent laboratory examinations, meeting diagnostic criteria for metabolic syndrome (*n* = 600). Crete (Greece), 2015. Diagnosed according to the National Cholesterol Education Program’s Adult Treatment Panel III (NCEP ATP III – revision 2005) criteria. Grey lines show the 95% Confidence Intervals (95% CIs).

Overall, 600 (73.6%; 95% CI 70.4, 76.6) patients in our sample were meeting the NCEP ATP III MetS criteria, with a non-significantly higher proportion being males (75.6%; 95% CI 71.0, 79.8) than females (72.0%; 95% CI 67.8, 76.0) (Table 3). Figure 3 presents data on the most frequently encountered risk factors among participants with MetS.

**Table 3.** Metabolic syndrome (MetS) risk factors in 815 primary care patients, 40 years of age or older, with availability of recent laboratory examinations. Crete (Greece), 2015.

MetS risk factors^a^	*n* (%; 95%CIs)^b^		
	Total	Males	Females
None	34 (4.2; 2.9,5.5)	7 (1.9; 0.9,3.8)	27 (5.9; 4.0,8.4)
1	72 (8.8; 6.9,10.8)	29 (8.0; 5.6,11.2)	43 (9.5; 7.0,12.4)
2	109 (13.4; 11.2,15.7)	52 (14.4; 11.1,18.3)	57 (12.6; 9.7,15.8)
3+ or MetS	600 (73.6; 70.4,76.6)	273 (75.6; 71.0,79.8)	327 (72.0; 67.8,76.0)

^a^According to the National Cholesterol Education Program’s Adult Treatment Panel III (NCEP ATP III–revision 2005) guidelines for metabolic syndrome (MetS).

^b^95% CIs, 95% confidence intervals.

## Discussion

### Main findings

The burden of MetS documented in this study reached 73.6%. Frequencies of CVD risk factors were 64.5% (males) and 61.1% (females) for hypertension, 69.3% (males) and 63.5% (females) for dyslipidaemia, 45.9% (males) and 36.5% (females) for diabetes and 21.2% (males) and 6.2% (females) for CHD. To the authors’ knowledge, this is the first study to report on MetS frequencies in primary care settings in Crete, Greece.

### Interpretation of study results

Several European studies have reported a significant MetS burden in primary care. A German study of adults visiting GPs found an age-standardised MetS prevalence of 18.7% (19.5% in males, 18.5% in females), according to NCEP ATP III [13]. In an Irish study of primary care patients aged 50–69 years, the NCEP ATP III MetS prevalence reached 20.7% (similar in males and females) [14]. A study conducted in a primary care setting in Spain indicated a burden of MetS of 29.2% in people over 45 years old [15]. A recent report from 10 European countries found a MetS prevalence of 24.3% with an age-associated increase in all its cohorts [16].

There are also several Greek studies reporting on MetS population frequencies, although it was not possible to track studies following exactly the same criteria and sampling methodologies as the present one. These include the MetS-Greece Multicentre Study of a representative sample of adults (>18 years old, NCEP ATP III criteria, prevalence: 23.6%) [17], MEDIS study involving elders (≥65 years old, NCEP ATP III criteria) from several islands (29%) [18] and ATTICA Study of adults (>18 years old, NCEP ATP III) from Athens (19.8%) [7]. Our findings report a higher MetS frequency from this sample of primary care practices in Crete; however, this data is not directly comparable to the above studies due to significant design and sampling differences (e.g. selective sample from a population of primary care patients 40 years of age and older with available clinical tests) have likely led to higher rates being documented. Furthermore, compared to the general population on adult residents, individuals seen in primary care practices in Greece tend to be older and have existing disease (ADD REF).

Another interesting finding concerns the trends related to the documented components of MetS criteria, relative to previously published data. Frequencies of elevated triglycerides, high fasting glucose and low HDL were remarkably higher in this study compared to the MetS-Greece study both when comparing with all general population and with the MetS diagnosed sub-sample (triglycerides: 70.3 vs. 25.7% general and 67.6% MetS, high fasting glucose: 61.8 vs. 18.2% general and 53.9% MetS, low HDL: 71.4 vs. 24.7% general and 54.3 MetS) [17]. This may reflect the fact that patients with elevated cholesterol may be more frequently seen in primary care, relative to the general population. The ATTEMPT study also reported high frequencies of hypertension (89.6% in males, 84.2% in females) and hypertriglyceridaemia (86.8% in males, 74.2% in females) in a sample of Greek outpatients [19].

Finally, this study identified important gender-related trends, with significantly larger proportions of males than females meeting NCEP ATP III MetS criteria. Gender analysis also documented significantly higher proportions of patients with hypertension, diabetes and triglycerides among males than females. In contrast, significantly higher proportions of patients with central obesity were documented among females. These gender-related trends in CVD risk factors have been previously reported in a study of 1501 Greeks with MetS [19]. Lower proportions of patients with hypertension among females may be attributed to beneficial effects of pre-menopausal hormones [20].

### Limitations

About half of the randomly selected GPs agreed to participate and this is a potential limitation for providing general conclusions. Furthermore, reasons for practice non-participation or withdrawal were not recorded. Although documenting physician response was not an aim of this study, it might have been that a missed opportunity to improve future patient recruitment practices led the participation of more organised or co-operative GPs a subsequently introduce a form of selection bias.

Since there are no data available regarding our reference population, sample size calculations were based on the general population of Crete. The sample size achieved was less than the targeted one. These may have led to a potential overestimation of the true MetS burden. Information about numbers of patients who refused participation was also not recorded.

The applied consecutive recruitment flow may have resulted in missing eligible patients, with implications for sample randomness and accurate identification of MetS frequencies. Additionally, not all GPs recruited the targeted number of patients. This results in potential bias increasing the likelihood that MetS would be detected (i.e. self-selection of participants by GPs), affecting sample representativeness and results’ generalizability. GPs not meeting the recruitment target were not excluded, since this was considered an enrolment completion defect rather than a gap of continuity.

This study was based on data available in GPs’ records, potentially introducing bias in terms of documentation practices. Although we conducted audits on consecutive patients over 40 years old, inclusion criteria required specific measurements to be on patient charts. Our sample is confined to a selected subpopulation of patients who have on their record the examinations required for MetS classification and who may have higher burden of MetS and other chronic conditions than the general primary care population. Given the number of participants that were excluded due to lack of test information on their records was not recorded, uncertainty is introduced regarding the true burden of MetS. Participants also had specific socio-demographic characteristics, including a fairly low level of education. The generalizability to other primary care settings is, therefore, unknown and should be taken into consideration when interpreting study findings.

### Study implications

Patients with MetS may have been over-represented in our sample by design; thus decisive conclusions cannot be derived regarding the actual MetS population frequencies. However, study findings agree with figures suggesting that Greece is facing what might be considered a ‘cardiovascular epidemic’ [21]. While Greece and Crete have been historically considered as a low-CVD risk population [22,23], a shift in the frequencies of risk factors and morbidity has been reported [24]. This may be partially attributed to changes in traditional dietary habits and lifestyle [25], along with increases in obesity and physical inactivity [26]. Reductions in CVD mortality are also occurring at a slower rate than in other European countries where educational and preventive measures have been more effectively applied and this should be a call to action [27].

Our findings should be understood within the contextual framework of severe economic recession that was prevailing in the country during study. Risk determinants are known to vary as a result of primary care quality and poorer lifestyle adoption due to socio-economic restrictions. The lack of selective cardiometabolic screening as standard of care within Greek primary care is also an issue that may impact MetS early detection and intervention. Available data suggests that the Greek financial crisis impacted preventive care-seeking, with healthy individuals visiting GPs less frequently than other countries and having fewer early detection opportunities [28,29]. Although study data were collected in 2015, evidence suggests that the cardiovascular profile of Greeks remains relatively stable or increased in the last two decades (1994–2016) [30], indicating that our results may still be valid today. However, further research is necessary to confirm this validity.

Findings of this primary care study add to evidence regarding the burden of MetS and CVD-risk factors in Greece. Cautiously interpreted, our data can be used to examine the practice gaps on prevention, early detection and treatment of MetS. The documented MetS burden warrants further investigation to overcome study limitations, validate trends, understand causes and inform future interventions.

## Conclusion

Our study has found a high prevalence of CVD risk factors and MetS among the sampled population of primary care patients in Crete with gender-related differences also documented. Study results should be interpreted in light of the study’s sampling methods and further research is needed to assess the magnitude of MetS in Greek primary care. Study findings, combined with published research and contextual factors, can be used to examine further preventive actions including awareness raising, enhancement of healthy lifestyle and promoting care integration.

## Supplementary Material

Supplementary Table
