# Supplementary material for: Burden of metabolic syndrome among primary care patients in Crete, Greece: A descriptive study
Source: Eur J Gen Pract. 2020 Dec 15;26(1):166–74. doi: 10.1080/13814788.2020.1851676 (PMC7744156; doi:10.1080/13814788.2020.1851676)
Supplement: Supplementary Table [file IGEN_A_1851676_SM3684.docx]

**Supplementary Table.** Categories of medication intake of 804 primary care patients, with or without cardiovascular diseases (CVDs) and without missing data on prescriptions. Crete (Greece), 2015.

|  | **Total** | | | **without CVDs** | | | **with CVDs** | | |
| --- | --- | --- | --- | --- | --- | --- | --- | --- | --- |
| **Medication categories** | **n** | **%** | **(95%CIs)^a^** | **n** | **%** | **(95%CIs)** | **n** | **%** | **(95%CIs)** |
| Hypertension | 485 | 64.8 | (61.3-68.3) | 335 | 60.4 | (56.5-64.5) | 150 | 77.7 | (71.9-83.6) |
| Coronary Heart Disease^b^ | 100 | 13.4 | (10.9-15.8) | 11 | 2.0 | (0.9-3.3) | 89 | 46.1 | (39.4-53.6) |
| Diabetes mellitus | 297 | 39.7 | (36.2-43.3) | 195 | 35.1 | (31.3-39.1) | 102 | 52.8 | (45.7-60.2) |
| Acute Myocardial Infraction | 3 | 0.4 | (0.01-0.9) | 0 | 0 |  | 3 | 1.6 | (0.01-3.4) |
| Atrial fibrillation, arrhythmias, angina, cardiovascular failure | 66 | 8.8 | (6.8-10.9) | 14 | 2.5 | (1.3-3.8) | 52 | 26.9 | (20.5-33.7) |
| Dyslipidemia (hypercholesterolemia, hypertriglyceridemia, both etc.) | 467 | 62.4 | (59.1-65.7) | 322 | 58.0 | (54.0-62.1) | 145 | 75.1 | (69.2-81.2) |
| Stroke | 10 | 1.3 | (0.5-2.3) | 1 | 0.2 | (0.01-0.7) | 9 | 4.7 | (1.6-7.9) |
| Venous failure, thrombosis | 5 | 0.7 | (0.1-1.3) | 1 | 0.2 | (0.01-0.7) | 4 | 2.1 | (0.5-4.3) |

^a^ 95%CIs: 95% Confidence Intervals (estimated based on bootstrap techniques).

^b^ Some medications were reported as “preventive”.
